# An atypical autistic phenotype associated with a 2q13 microdeletion: a case report

**DOI:** 10.1186/s13256-018-1620-4

**Published:** 2018-03-18

**Authors:** Jokthan Guivarch, Clarisse Chatel, Jeremie Mortreux, Chantal Missirian, Nicole Philip, François Poinso

**Affiliations:** 1 Department of Child Psychiatry, Aix-Marseille University, APHM, Sainte Marguerite Hospital, Marseille, France; 2 PACA Autism Resource Center, APHM, Sainte Marguerite Hospital, Marseille, France; 3 Department of Medical Genetics, Cytogenetics Laboratory, APHM, Timone Hospital, Marseille, France; 4 Department of Medical Genetics, Referral Center for Developmental Anomalies, Aix-Marseille University, APHM, GMGF, Timone Hospital, Marseille, France

**Keywords:** 2q13 deletion, Autism spectrum disorder, Atypical autistic phenotype

## Abstract

**Background:**

Autism spectrum disorders are serious neurodevelopmental disorders that affect approximately 1% of the population. These disorders are substantially influenced by genetics. Several recent linkage analyses have examined copy number variations associated with autism risk. Microdeletion of the 2q13 region is considered a pathogenic copy number variation. This microdeletion is involved in developmental delays, congenital heart defects, dysmorphism, and various psychiatric disorders, including autism spectrum disorders. There are only 34 reported cases with this chromosomal deletion, and five cases of autism spectrum disorders have been identified among them. The autistic phenotype associated with this microdeletion has never been described.

**Case presentation:**

We describe the case of a 44-month-old Caucasian girl with the 2q13 microdeletion and autism spectrum disorders with global development delay but no associated organ anomalies. We examined the autistic phenotype using different workups and observed an atypical phenotype defined by relatively preserved relational competency and imitation abilities.

**Conclusions:**

The main contribution of this case report is the precise description of the autistic phenotype in the case of this deletion. We observed some atypical clinical features that could be markers of this genetic anomaly. We have discussed the pathophysiology of autism associated with this microdeletion and its incomplete penetrance and variable expressivity.

## Background

Autism spectrum disorders (ASD) are serious neurodevelopmental disorders affecting 1% of the population [1, 2]. According to the *Diagnostic and Statistical Manual of Mental Disorders*, *Fifth Edition* (DSM-5), these disorders manifest as a dyad of symptoms, consisting of persistent deficits in social communication and social interaction (SI) in addition to restricted and repetitive patterns of behavior, interests, or activities [1]. Although the pathophysiology of these disorders is not yet known, ASD are considered neurodevelopmental disorders that are influenced by genetics [3–5].

### Autism and genetics

The prevalence of ASD among siblings of affected probands is estimated at 8–19% [6]. Previous studies conducted in twins suffering from ASD revealed a concordance rate of 80–90% in monozygotic twins and 20% in dizygotic twins [7]. Autism may be a disease with complex heredity [3, 6]. Thus, the phenotype could be the result of “a complex system of expression including genetic, epigenetic, environmental [8] and/or stochastic factors” [3]. Genetic anomalies may impact synaptic functioning by interfering with the establishment of functional neuronal networks through alterations in neuronal apoptosis, protein synthesis, or even the activity of neuroligins and neurexins [9, 10].

Devlin and Scherer have defined four groups according to the type of genetic involvement. These four groups are syndromic autism (for example, Fragile X), chromosomal anomalies, rare penetrant genes, and rare copy number variations (CNVs) [6]. CNVs are genomic rearrangements that are undetectable by karyotype analysis; however, they can be identified by array comparative genomic hybridization (aCGH) analysis [6]. *De novo* or inherited CNVs (for example, 2q13 microdeletion) have been identified in up to 10% of cases of autism [6, 11, 12].

### The 2q13 deletion

The 2q13 region contains clusters of segmental duplications, which facilitate non-allelic homologous recombination and can lead to recurrent deletions and duplications [13].

A recurrent 1.71 Mb microdeletion [12–14] has been reported in 34 patients [12–21], five of whom have ASD [13, 16, 21]. The deletion is inherited from healthy parents in a significant number of cases and, as a result, its clinical significance was initially unclear. However, this CNV is now considered pathogenic and shows variable penetrance [13, 16, 21].

The clinical phenotype is variable, as are the reasons for referral (14, 16, 20, 21). Mild dysmorphic facial features have been reported in the majority of cases. However, there are no patient pictures available, and the typical facial gestalt has not been described. The patients may show either macrocephaly or microcephaly. Congenital heart defects (CHDs) are the most frequent congenital malformations. Several other congenital defects have been described in single cases, including scoliosis and renal and genital malformations. In addition, developmental delays, hypotonia, and disorders in fine and global motor skills, such as balance and coordination, are widely reported. There are also cases of attention deficit/hyperactivity disorder linked to this microdeletion [13, 16, 20, 21].

Among the five patients with 2q13 microdeletion suffering from autistic disorders [13, 16], two were described as having ASD and two as having pervasive developmental disorder not otherwise specified (PDD-NOS). There was no explanation of the reasons that led to a diagnosis of PDD-NOS. We had no information regarding the fifth case.

The precise autistic phenotype in the case of a 2q13 microdeletion has never been described.

We report the case of a 44-month-old (3 years and 8 months) child with an interstitial 2q13 microdeletion who suffered from atypical ASD. We describe the autistic phenotype with standardized tools.

## Case presentation

### Clinical elements

This case report describes a 44-month-old (3 years and 8 months) Caucasian girl who was the second child of healthy and non-consanguineous parents. The family history was negative. She was born at term after an uncomplicated pregnancy and delivery, and the birth parameters were normal.

At 6 months of age, her parents noted behavioral anomalies, including avoidance of gaze, absence of turning when called by her name, and retreating into repetitive, self-centered games. In addition, she had delayed gross motor development. She began sitting at 12 months and walking at 19 months of age. Her medical history was otherwise unremarkable except for recurrent episodes of bronchiolitis. The day care center staff noticed socialization difficulties and the appearance of motor mannerisms with rocking back and forth. Thus, ASD was suspected. At 2 years and 8 months of age, she was referred to a referral center for developmental problems because the pediatrician suspected Angelman syndrome. A clinical examination showed facial dysmorphism with coarse features, full cheeks, anteverted nares, and a large mouth. Her height and weight were in the normal range, with a head circumference (HC) at −1 standard deviation (SD). A neurological examination was normal.

When she was 3 years and 8 months old, a multidisciplinary diagnostic evaluation was conducted at a referral center (PACA Autism Resource Center) using standardized tools. A global psychomotor delay was found, with a developmental age of 23 months in fine motor skills and 27 months in overall motor skills. In addition, subtle global hypotonia was also noted. An electroencephalogram (EEG) and cerebral magnetic resonance imaging (MRI) with spectroscopy showed no anomalies. An ear, nose, and throat (ENT) workup revealed physiological audition.

The diagnosis was performed according to the DSM-5 criteria with the help of the following multidisciplinary workups and standardized tools (refer to Table 1): the Autism Diagnostic Observation Schedule, Second Edition (ADOS-2) [22]; the Autism Diagnostic Interview-Revised (ADI-R) [23]; and the Vineland Adaptive Behavior Scales, Second Edition (VABS-II) [24].

**Table 1:** The autism scales

ADOS-2 in French [22] (module 1):	
• Social affect, 17 (communication, 4; reciprocal social interaction, 13)	
• Restricted and repetitive behaviors, 5	
• Overall total, 22 (ASD threshold, 11; autism threshold, 16)	
• ADOS-2 comparison score, 7 (moderate)	
ADI-R [23]:	
• Reciprocal social interactions, 18 (autism threshold, 10)	
• Nonverbal communication, 10 (threshold, 7)	
• Repetitive behaviors and stereotyped patterns, 7 (threshold, 3)	
• Developmental anomalies at or before 36 months, 5 (threshold, 1)	
Vineland-II/VABS-II [24] standard scores:	
• Communication, 32	
• Daily life, 38	
• Socialization, 42	
• Motor skills, 50	
• Composite score of adaptive behavior, 26	

*ADI-R* Autism Diagnostic Interview-Revised, *ADOS-2* Autism Diagnostic Observation Schedule, Second Edition, *ASD* Autism spectrum disorder, *VABS-II* Vineland Adaptive Behavior Scales, Second Edition

The results of ADOS-2 module 1 led to the diagnosis of autism with a moderate level of symptomatology. She was nonverbal and was tested with module 1, which is the specific module for preverbal children. During the administration of the ADOS-2, we noticed that gesturing was poor and response to her name was lacking. She had no spontaneous initiation of joint attention. However, she was able to have directed vocalizations and presented a large variety of directed facial expressions. Her eye contact was of acceptable quality. There was a shared pleasure observed in the interactions, and she presented directed smiles.

The results of the ADI-R showed scores greater than the cut-off for autism in the three domains, with developmental anomalies noted before 36 months. It should be noted that the ADI-R was performed even though she presented a global level of development lower than 24 months, and the results contributed to the diagnostic assessment.

The VABS-II evaluates adaptive behavior. The test result showed a low composite score of adaptive behavior (standard score 26). Adaptation was low in all domains, namely, motor, socialization, daily life, and communication.

Her communication competencies were evaluated using the Early Social Communication Scales (ESCS), which allows evaluation of development in three domains of early communication [25]. Her level of SI was similar to that of an 18-month-old child.

Her level of conjoined attention (CA) was 13 months. Her regulation of behavior was equivalent to 12 months. Overall, this child presented a social communication development level of 14 months.

Her level of development was evaluated using the Psychoeducational Profile, Third Edition (PEP-3) [26]. The results revealed overall delay. Her verbal and preverbal cognition levels were at 16 months.

Her receptive and expressive language level corresponded to that of a child younger than 12 months of age. Her developmental age was 19 months in global motor skills. She does not present any clear lateralization and has difficulties with static equilibrium. Her developmental age was 20 months for fine motor skills. She cannot coordinate her two hands and has difficulties grasping objects with two fingers. However, she spontaneously engages in scribbling. Her oculomotor imitation was similar to that of a 22-month-old child. She can engage in imitation through the manipulation of objects and actions. However, her imitation of gestures and sounds is weaker.

The overall result of the tests indicated ASD associated with developmental delays. Her strengths included imitation and interactions with other people through gaze and smiling.

### Genetic analyses

Genetic analyses were conducted at the referral center for developmental problems. The pediatrician initially suspected Angelman syndrome due to the developmental delay and frequent smiling. However, analysis of chromosome 15 methylation invalidated this hypothesis and excluded 15q11.2 microdeletion or uniparental chromosome 15 disomy.

The aCGH was performed using a 4 × 180 K whole-genome oligonucleotide microarray following the manufacturer’s protocol (Agilent Technologies, Santa Clara, CA, USA). The results were interpreted with Cytogenomics software v3.0.1.1 (ADM2 method). A CNV was considered if at least three contiguous oligonucleotides presented an abnormal mean log ratio (> 0.25 or ≤ 0.25). This analysis identified a 1.7 Mb interstitial *de novo* microdeletion in chromosomal region 2q13 (on the long arm of chromosome 2) between proximal chromosomal position 111,399,243 and distal position 113,102,535 (hg19/GRCh37). Using the International System for Human Cytogenetic Nomenclature 2016, the deleted region identified by the microarray analysis was designated as follows: arr[GRCh37] 2q13(111399243_113102535)× 1 dn. The region encompasses the following nine genes: *BUB1*, *ACOXL*, *BCL2L11*, *MERTK*, *FBLN7*, *TMEM87B*, *ANAPC1*, *ZC3H8,* and *ZC3H6*. Fluorescent *in situ* hybridization (FISH) analysis with probes RP11-438 K19 confirmed this rearrangement. Previous studies reported that recurrent CNVs of this chromosomal region are implicated in the 2q13 microdeletion/microduplication syndrome [12, 13].

## Discussion and conclusions

### Discussion of the “atypical” phenotype

Our young patient presents with DSM-5 diagnostic criteria for ASD. However, we observed some clinical atypia in cognitive abilities, motor functioning, and social adaptation. We found the following features in our patient:

- During the administration of the ADOS-2, a possibility of being in a relationship with others by gaze and by regular directed smiles was noted. In addition, she shared her pleasure and displeasure rather easily despite being nonverbal.
- The VABS-II indicated that her profile based on standard scores is the following: Motor skills > Socialization > Daily life > Communication. The profile of classically nonverbal children with ASD is different and tends to show the following results: Motor skills > Daily living > Socialization > Communication [24]. Our patient has better socialization abilities than daily life skills, which is atypical.
- The PEP-3 development workup revealed global delay, which suggests a very probable intellectual deficit in this child. The development levels in the PEP are considered good indicators of cognitive functioning in both verbal and nonverbal children [27]. Our patient workup revealed that her oculomotor imitation was better than her global and fine motor skills, while the majority of children with ASD habitually have more difficulties in the domain of imitation than in motor skills [28]. The activities of imitation involving looking at the interlocutor, requesting his or her attention, expressing a request for maintaining an activity, and expressing pleasure and displeasure are all activities that she accomplished despite her ASD.
- Her profile in the ESCS was similar to the other test results. The three domains of early social communication are CA, SI, and behavior regulation (BR). Interestingly, the same profile is observed in children with ASD with or without intellectual deficit: BR > SI > CA [29]. However, our patient displays a different profile, namely, SI > CA > BR. Thus, even though she presented a delay in the different domains of social communication, the delay is less significant with respect to SIs.

In the literature, there are only five cases of Autistic disorders with 2q13 microdeletion – including two cases of PDD-NOS [16] – without description of autistic phenotype in this deletion. The two cases of children diagnosed PDD-NOS may have an autistic phenotype similar to that of our patient. PDD-NOS is a diagnosis from the previous version of the *DSM*, the *Fourth Edition text revision* (DSM-IV-TR) [30], and was used when the complete criteria for a pervasive development disorder were not found. This diagnosis could have been selected because of the relational abilities of these children.

Thus, autism with atypia could also be part of the phenotype associated with a 2q13 microdeletion.

### Discussion of the genes involved

The dysmorphism and HC anomaly generally observed in cases with this deletion could involve the *FBLN7* gene, as suggested by experiments conducted in zebrafish. The *FBLN7* gene encodes the protein fibulin-7, which is a glycoprotein that interacts with cells through integrins, and this protein plays a role in the embryonic differentiation of odontoblasts and in the formation of facial bones [20].

There are limited data available for the *TMEM87B* gene, but its transcripts are found in adult and fetal tissues, including cardiac and cerebral tissue [20]. Thus, that gene could also be involved in developmental delays, autism disorders, and other psychiatric phenotypes.

Previous experiments have shown that the *FBLN7* and *TMEM87B* genes are both involved in CHDs and that the most serious CHDs occur when both genes are lost in the context of a microdeletion [20, 21].

The *BCL2L11* gene could be involved in the neurodevelopmental disorders observed in association with this microdeletion [13]. The gene codes for the protein BCL2L11, which is a Bcl2-like apoptotic protein and may be implicated in neuronal apoptosis [13, 31]. The downregulation of Bcl2 has been found in the frontal and parietal cortex and the cerebellum in cases of ASD [32, 33]. The loss of a Bcl2-like protein could alter neuronal apoptosis and may be involved in autism [16, 33] by interfering with the establishment of functional neuronal networks, which is consistent with the current neurobiological concept of autism [9].

The *ACOXL* gene could also be involved in neurodevelopmental disorders according to previous studies by altering the metabolism of fatty acids [16].

It is currently unclear why parents of affected children, who are carriers of the microdeletion, are asymptomatic or only mildly symptomatic. The incomplete penetrance and variable expressivity can complicate genetic counseling [13, 21].

Hladilkova *et al*. suggested that break points around the deletion that are variable from one patient to another may explain the incomplete penetrance [13]. This possibility implies that there are different genes deleted from the chromosome segment. It is also possible that loss of heterozygosity through the microdeletion could reveal a recessive allele [13].

Both Hladilkova *et al*. and Riley *et al*. supported Girirajan and colleagues’ theory of “the second hit,” which would amplify the phenotype [13, 21, 34]. The individuals who carry the microdeletion may have other CNVs that contribute to or modify the phenotype [13, 21, 34]. Yu *et al*. and Hladilkova *et al*. found this result in certain cases of 2q13 microdeletions [13, 16]. The second hit could also correspond to an environmental factor influencing the phenotype [13, 34]. Thus, the second-hit theory suggests that asymptomatic carriers of this microdeletion would not present any other genomic imbalance that could reveal the pathogenicity of these deletions [34].

## Conclusion

We have described the case of a child with a 2q13 microdeletion, who suffers from ASD with atypia and a developmental delay. The main contribution of this case report is the precise description of the autistic phenotype in the case of this deletion. We have observed an atypical phenotype defined by relatively preserved relational competencies and imitation abilities. This autistic phenotype could be found in other cases of 2q13 microdeletion. Additional cases have to be reported and compared with our observation to confirm our findings.

This case reveals the indication for microarray analysis in cases of ASD without severe developmental delay, congenital malformation, or clear dysmorphic features. Obtaining a genetic diagnosis is important for proper management of the patient and genetic counseling for the family. The identification of a chromosomal microdeletion could enable prevention of comorbidities in children with ASD through knowledge of the deleted chromosomal region and the genes contained in the lost region [21]. It could also improve the genetic counseling provided to parents for later pregnancies. Systematic genetic testing for children with ASD is conducted at certain specialized universities with a diagnostic success rate of 35–40% [3].

We do not support the generalization of genetic testing because variants of unknown significance could be discovered. In addition, as aCGH investigates chromosomal imbalances throughout the genome, there is a possibility of identifying unexpected findings not related to the indication of the test. Such variants may have potential health or reproductive significance for the patient or his/her family, raising difficulties in genetic counseling and presenting an ethical issue as illustrated by Lefebvre *et al*. in a 7-year French national survey [35]. CNVs affecting tumor suppressor genes cause a predisposition to cancer [36]. Charcot–Marie–Tooth type 1 and hereditary neuropathy with liability to pressure palsy (HNPP), linked to reciprocal duplication and deletion, respectively, of the 17p region, as well as autosomal dominant polycystic kidney disease and neurofibromatosis type 1, are a few examples of autosomal dominant disorders that can be identified by aCGH. CNVs can be inherited, leading to the diagnosis of an unsuspected disease in a parent. Chromosome X deletion in a girl can reveal carrier status for an X-linked condition. This finding has no clinical utility for the proband but raises the question of genetic counseling for the family [37]. Before disclosing such information, the clinician must evaluate the balance between potential harm and benefit for the patient or his/her family. The possibility of secondary findings and their possible consequences should be discussed during the pretest counseling session and explicitly mentioned on the informed consent form [36]. It is important to rationalize the request for genetic testing. In France, genetic testing is requested in cases of syndromic autism (with dysmorphia, organ anomalies, or anomalies in the neurological pediatric examination). It would be appropriate to broaden the indications in cases of autism with atypia as seen in the present case.

## Abbreviations

aCGH: Array comparative genomic hybridization
ADI-R: Autism Diagnostic Interview-Revised
ADOS-2: Autism Diagnostic Observation Schedule Second Edition
ASD: Autism spectrum disorder
BR: Behavior regulation
CA: Conjoined attention
CHD: Congenital heart defect
CNV: Copy number variation
DSM-IV-TR: *Diagnostic and Statistical Manual of Mental Disorders Fourth Edition* text revision
DSM-5: *Diagnostic and Statistical Manual of Mental Disorders Fifth Edition*
EEG: Electroencephalogram
ENT: Ear nose and throat
ESCS: Early Social Communication Scales
FISH: Fluorescent *in situ* hybridization
HC: Head circumference
HNPP: Hereditary neuropathy with liability to pressure palsy
PDD-NOS: Pervasive developmental disorder not otherwise specified
PEP-3: Psychoeducational Profile Third Edition
SD: Standard deviation
SI: Social interaction
VABS-2: Vineland Adaptive Behavior Scales Second Edition
